# Dual-Quantum-Dots-Labeled Lateral Flow Strip Rapidly Quantifies Procalcitonin and C-reactive Protein

**DOI:** 10.1186/s11671-016-1383-z

**Published:** 2016-03-25

**Authors:** XiaoPing Qi, YunYe Huang, ZhongShi Lin, Liang Xu, Hao Yu

**Affiliations:** Shenzhen Sixth People’s Hospital, Shenzhen, 518052 People’s Republic of China; Biomedical Engineering, Shenzhen Polytechnic, Shenzhen, 518055 People’s Republic of China; Shenzhen Institute for Drug Control, Shenzhen, 518057 People’s Republic of China

**Keywords:** Quantum dots, Lateral flow strip, Procalcitonin, C-reactive protein, Blood

## Abstract

In the article, a dual-quantum-dots-labeled (dual-QDs-labeled) lateral flow strip (LFS) method was developed for the simultaneous and rapid quantitative detection of procalcitonin (PCT) and C-reactive protein (CRP) in the blood. Two QD-antibody conjugates with different fluorescence emission spectra were produced and sprayed on the LFS to capture PCT and CRP in the blood. Furthermore, a double antibody sandwich method for PCT and, meanwhile, a competitive inhibition method for CRP were employed in the LFS. For PCT and CRP in serum assayed by the dual-QDs-labeled LFS, their detection sensitivities reached 0.1 and 1 ng/mL, respectively, and their linear quantitative detection ranges were from 0.3 to 200 ng/mL and from 50 to 250 μg/mL, respectively. There was little evidence that the PCT and CRP assays would be interfered with each other. The correlations for testing CRP and PCT in clinical samples were 99.75 and 97.02 %, respectively, between the dual-QDs-labeled LFS we developed and commercial methods. The rapid quantification of PCT and CRP on dual-QDs-labeled LFS is of great clinical value to distinguish inflammation, bacterial infection, or viral infection and to provide guidance for the use of antibiotics or other medicines.

## Background

C-reactive protein (CRP) is a well-established acute phase reactive protein that is synthesized by hepatic cells when there is an invasion by microorganisms in the human body, or there is tissue damage. Elevated CRP is a promising indicator of sepsis in neonates and other age groups. Detection of high concentration of CRP is of comprehensive clinical significance in the diagnoses of severity, progress, prognosis, and therapeutic effect of infection and cancers [26]. CRP concentration in the serum of normal people is lower than 10 μg/mL. It indicates a risk of infection when the CRP concentration is higher than 15 μg/mL and severe infection when CRP concentration reaches 200 μg/mL [24].

Procalcitonin (PCT) is a recently found endogenous, non-steroidal, and anti-inflammatory material that is produced when the human body is induced by bacterial endotoxin when there is a bacterial infection [9]. Under normal physiological conditions, PCT concentration in C cells of the thyroid is so low that it can hardly be detected. Bouadma et al. [4] have shown that reference indications of PCT served as a biomarker for the detection of bacterial infection. In the blood of normal people, PCT concentration is lower than 0.25 ng/mL. But it is from 0.25 to 0.5 ng/mL for the people with mild or local bacterial infection, and from 0.5 to 1 ng/mL for the people who have a chance of being infected or having septicemia and thus need dynamic observation, and from 1 to 100 ng/mL for the people who suffer from systematic bacterial infection and thus should be closely monitored as well as given specific anti-infective therapy and supportive treatment when necessary.

Despite of other potential discriminatory biomarkers such as IL-6 [7], simultaneous and rapid detection of CRP and PCT can be helpful in (i) differentiating bacterial infection from non-infective causes of inflammation; (ii) differentiating acute from chronic bacterial infection as well as local from systematic bacterial infection; (iii) and furthermore, determining the appropriate dosage and duration of antibiotic therapy [12, 23, 24, 29]. Recently, researches showed that the combination of PCT and CRP can help assess hematopoietic stem-cell transplant [1]; distinguish candidemia from bacterial sepsis [11]; also assess community-acquired pneumonia in children [2].

So far, CRP and PCT concentration can be semi-quantitatively detected by lateral flow strip (LFS). Methods for quantitative detection of CRP include enzyme-linked immunosorbent assay (ELISA) [27], rate immuno-nephelometry [10], and latex-enhanced immunoturbidimetric [15]. Methods for quantitative detection of PCT include immunofluorescence [17, 21] and immunochemiluminescence [14, 19]. The above-mentioned methods by which quantitative detection can be conducted are expensive owing to both equipment and detection process, and they are also complicated and time-consuming. By contrast, detection by LFS is rapid, simple, and relatively cheap, but the results are semi-quantitative due to its low detection sensitivity.

Quantum dots (QDs), fluorescent semiconductor nanoparticles introduced to biomedical research nearly two decades ago [5, 6], have received attention in recent years because of a number of attractive features: wide range of excitation wavelength, narrow and symmetric spectral peak in its emission spectra, little external interference, and high stability [13, 18, 20, 28]. Some literatures established the QDs-labeled LFS to rapidly and quantitatively detect traces of the biomarkers in the blood or other fluids without pretreatment [3, 8]. To further utilize the advantages of QDs, a dual-QDs-labeled LFS method was introduced in our article for the simultaneous quantification of both CRP and PCT in human blood. In this method, two QD-antibody conjugates with different fluorescence emission spectra were produced and strayed on the LFA. A double antibody sandwich method for PCT, meanwhile a competitive inhibition method for CRP is adopted in the LFS assay, to minimize non-specific adsorption and the hook effect. The characteristics of QDs were measured. The characteristics of dual-QDs-labeled LFS method, such as the sensitivities, linear quantitative ranges, linearities, interferences, stability, and correlations with existing methods, were analyzed for PCT and CRP assays, respectively.

## Methods

### Materials

Goat anti-mouse IgG, mouse monoclonal CRP antibody (CRP135 mAb), mouse monoclonal PCT antibody (14C12 mAb), its paired mouse monoclonal calcitonin antibody (14A2 mAb), CRP-free serum, and PCT-free serum were obtained from Hytest. Analytically pure reagents: cadmium oxide (CdO), selenium powder (Se), powdered sulfur (S), oleic acid (OA), zinc oxide powder (ZnO), octadecene (ODE), octadecyl amine (ODA), trioctylphosphine oxide (TOPO), sodium azide were obtained from Sigma-Aldrich; mercaptoacetic acid (MAA), trichloromethane, normal hexane, methanol, acetone, heptane, glycine, sodium tetraborate, and sucrose were from China National Pharmaceutical Group. Standard human CRP and standard human PCT, bovine serum albumin (BSA), and phosphate-buffered saline (PBS) were from Sigma-Aldrich. Guarantee reagents: N-hydroxysuccinimide (NHS) and carbodiimide (EDC) were from China National Pharmaceutical Group. Nitrocellulose membrane and glass cellulose membrane were from Millipore; the whole blood separator was from Shanghai JieYi Biotechnology; and the stickiness baseplate was from Shanghai Liangxin Science and Technology Ltd. Ultrapure water (Milli-Q) is used in the detections.

### Synthesis of QDs

Preparation for hydrophobic CdSe/ZnS QDs is as described in reference [22]. Briefly, sulfur precursor is prepared by successively adding 20 mL ODE and 0.06 g S powdered into the flask and heating them at 150 °C under nitrogen flow. The zinc precursor is prepared by successively adding 0.16 g ZnO powder, 5.64 g OA, and 13.7 mL ODE into the flask and heating them at 310 °C under nitrogen flow. Four milliliters of ODE, 0.4 mL OA, and 0.3 M CdO are put into a flask and heated to 300 °C. Next, the solution is cooled to room temperature, and then 2.5 g ODA and 0.5 g TOPO are added into the flask. After the solution is heated at 280 °C, 1.8 mL ODE solution with 1.8 M Se is injected into the flask. The CdSe nanocrystals are grown at 260 °C, and then centrifuged at 12,000 rpm for 15 min, separated and preserved in hexane.

Three milliliters of ODE and 1 g ODA are loaded into a flask followed by adding the synthesized CdSe nanocrystals (preserved in hexane). Heating them in 100 °C under the nitrogen protection until the hexane is completely removed. Sulfur (0.52 mL) and zinc precursor are slowly and successively added, and the temperature is increased to 180 °C and kept for 10 min. And then 0.77 mL sulfur and zinc precursor are slowly and successively added, and the temperature is increased to 200 °C and also kept for 10 min. In accordance to the differences of growth layers, different volumes of sulfur and zinc precursor are added alternately. The final reaction temperature is kept at 240 °C, and circulation flux is kept for 1 h. The obtained CdSe/ZnS QDs is repeatedly purified by adding 10 mL hexane and extracting wastes with methanol, and then dispersed for use in a small amount of chloroform.

### Phase Transfer of QDs

The brief method of synthesizing hydrophobic CdSe/ZnS QDs into water soluble is as in reference [16] with little modification: the hydrophobic CdSe/ZnS QDs is first precipitated with acetone and then redispersed in trichloromethane. After that, MAA is added to the solution. After intensive mixing and standing, the mixture was stored solution at room temperature for at least 2-h reaction, until the mixture solution became opaque which indicates the formation of MAA-QDs. The MAA-QDs are then centrifuged out at 10,000 rpm for 10 min. Soft pellet (100–200 μL) is collected and re-suspended in ultrapure water. The above-mentioned operations are repeated for two to three times. By the dropwise addition of 1 mol/L NaOH solution, the pH is adjusted to 10–11. MAA-QDs are precipitated by adding acetone and water (50:50 *v*/*v*) to the aqueous solution. The precipitation is slowly dried overnight under mild vacuum and then slowly re-dispersed under mild agitation in pH 7.2 0.1 mol/L PBS with the final concentration of 1 μmol/L.

### Preparation of QD-Antibody Conjugates

Antibodies were attached to the surface of QDs by chemical crosslinking to obtain QD-antibody conjugates. The brief procedures are as follows: 60 pmol of QDs, 10 μg of EDC, 15 μg of NHS solution, and 20 μg of CRP135 mAb solution are added into PBS. And then, the solution is mixed evenly; the mixed solution is shaken gently for 24 h at room temperature; after that, 1 mg of glycine for blocking is added in the solution. The mixture is further separated and purified by using an ultra-filtration membrane (Microcon YM30, Millipore) to obtain QD-CRP135 mAb conjugates. Finally, the purified QD-CRP135 mAb conjugates is dissolved in PBS and stored at 4 °C for further use. QD-14C12 mAb conjugates can be obtained by the same way. The fluorescence emission spectra and quantum yields of QD-antibody conjugates and hydrophobic CdSe/ZnS QDs were acquired with the ultraviolet-visible (UV-Vis) spectrophotometer (Shimadzu 2450, Shimadzu) and the fluorescence spectrometer (Ls55, PerkinElmer).

### Preparations for LFS

The LFS consists of several key components, such as a sample pad, a filter pad, a conjugate pad, a chromatographic membrane, an absorbent pad, and a baseplate. The QD-CRP135 mAb conjugates and the QD-14C12 mAb conjugates were evenly sprayed onto a pad to make a conjugate pad by XYZ3000 (Bio-Dot). 14A2 mAb was sprayed onto the processed chromatographic membrane to form detection line 1, CRP was fixed to form detection line 2 and goat anti-mouse IgG was fixed to form the quality control line. The detection lines and quality control line are 1.5-mm-wide with 4 mm of space between two different lines. After the pads were laminated, the LFS was cut vertically with a slitter into 5-mm wide, sealed in dry bags and stored at 4 °C.

### Design of the Fluorometry System

**Fig. 1:**
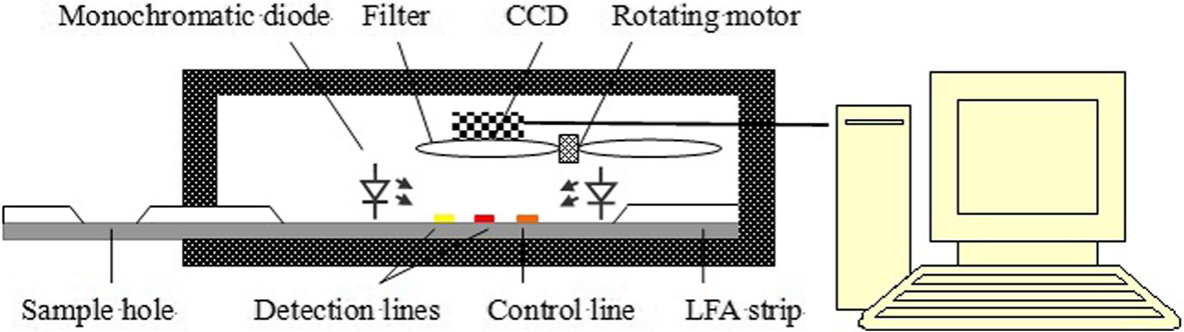
The schematic structure of fluorescence measuring instrument

In this paper, the self-designed fluorometry system was established, whose elementary structure is shown in Fig. 1. The system consists of highlight monochromatic diodes as the excitation light source. The monochromatic excitation light irradiates the PCT detection line, the CRP detection line, and the quality control line on the LFS. QDs combined on them stimulate different maximum emission spectra, which are received by CCD after going through narrow band filters. The CCD is cooled by a semiconductor freezer and stored at approximately 10 °C to reduce as much dark current noise as possible. The emission fluorescence intensity of QDs combined by PCT and CRP is detected by two different narrow band filters which are controlled and rotated by the electric rotating motor. The corresponding CRP and PCT concentration data are output after a matching analysis with the computer’s standard curve.

### Standard Sample Assay

To produce the standard samples, different amount of PCT (0–200 ng/mL) were added into the PCT-free serum solution, as the CRP concentration in the mixture was fixed at 5 ng/mL, 50 ng/mL, 500 ng/mL, 5 μg/mL, and 50 μg/mL separately. Similarly, different amounts of CRP (0–250 μg/mL) were added into the CRP-free serum, as the PCT concentration in the mixture was fixed at 5, 50, and 500 ng/mL separately. At room temperature, 100 μL of standard solution was added onto the sample pad of the LFS for the reaction. After 15 min of vertical standing, the LFS was inserted into the self-designed fluorescence detection device, and the fluorescence intensity from QDs was assayed on detection lines 1 and 2 and the control line. Every standard sample was detected five times, and the mean value of the results was taken.

### Clinical Samples Assay

A total of 54 human blood samples from patients with bacterial infections and from normal people were obtained from Shenzhen Sixth People’s Hospital. Each clinical sample was divided equally into two portions. In one portion, its CRP concentration was detected by ELISA and its PCT concentration was detected by immunofluorescence method by hospital. In the other portion, its CRP and PCT concentrations were detected by dual-QDs-labeled LFS method established in this paper. Each sample was detected three times respectively, and the mean value of the results was taken. Patient consent was not obtained as all personal identifiers and patient information were delinked from the specimens.

## Results

### Characterization of the QDs

**Fig. 2:**
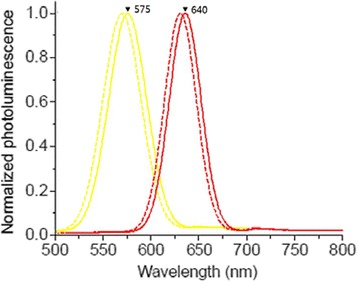
The maximum emission wave of QD-CRP135 mAb conjugates was 575 nm, and the maximum emission wave of QD-14C12 mAb conjugates was 640 nm (*dashed line*: before modification, *solid line*: after modification)

Fluorescence emission spectra before and after the QDs are modified are shown in Fig. 2. As it shows, there were slight redshifts at the fluorescence emission peaks of QDs after they were modified by antibodies. The maximum fluorescence wavelengths emitted were, respectively, 575 and 640 nm with the corresponding peak width at half height of 40 and 30 nm. The fluorescence emission peaks were narrow and approximately symmetrical. Thirty-four percent quantum yield (QY) was recorded for QD-CRP135 mAb conjugates in reference to Cresyl Violet Acetate (Sigma-Aldrich) in methanol, and 36 % QY recorded for QD-14C12 mAb conjugates, as compared to 55 % QY recorded for hydrophobic CdSe/Zn QDs in chloroform.

### Principles and Observations of Dual-QDs-Labeled LFS

To overcome the large difference of content ranges between PCT (ng/mL) and CRP (μg/mL) in the blood to be assayed, in this paper, the LFS assay developed was based on double antibody sandwich method for the detection of PCT and meanwhile competitive inhibition method for the detection of CRP [25]. By this way, it can minimize non-specific adsorption and the hook effect that is likely to occur when the sample concentration is relatively high.

**Fig. 3:**
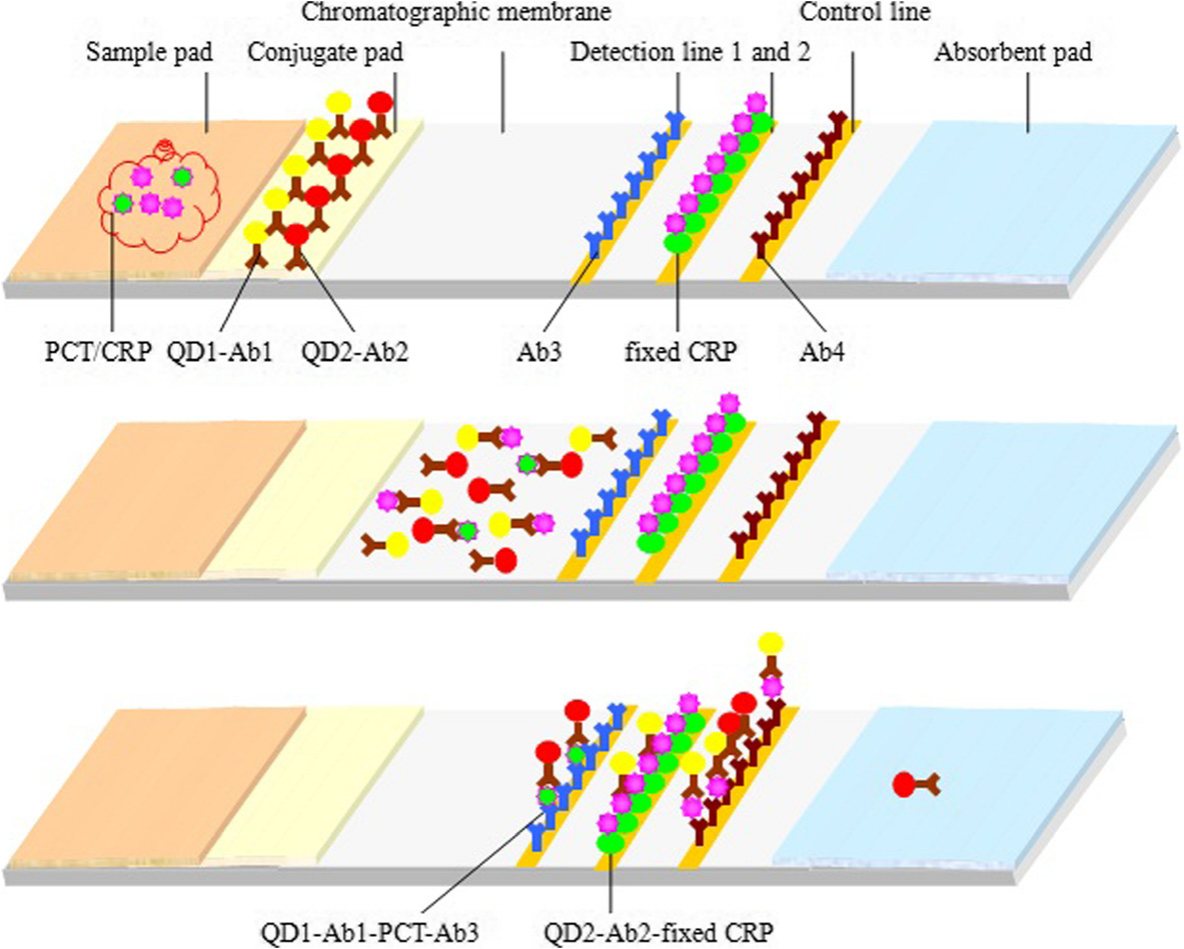
The principle of dual-QDs-labeled LFS testing PCT and CRP simultaneously

**Fig. 4:**
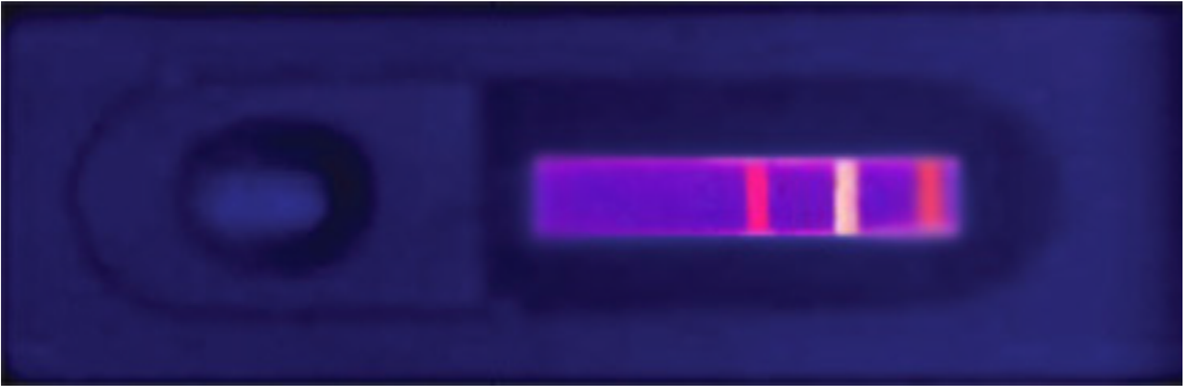
Direct observation of the strip added with 20 ng/mL PCT and 2 μg/mL CRP

The specific detection principles are shown in Fig. 3. When the sample is added onto the sample pad, it rapidly drenches the conjugate pad and dissolves QD-14C12 mAb conjugates (QD1-Ab1 for short) and QD-CRP135 mAb conjugates (QD2-Ab2 for short). If target PCT exists in the sample, the QD1-Ab1-PCT immunocomplex is synthesized; meanwhile, if a target CRP exists, also the QD2-Ab2-CRP is synthesized. Then, the sample contains QD1-Ab1-PCT, extra QD1-Ab1 unreacted, QD2-Ab2-CRP, extra QD2-Ab2 unreacted, and other components in the blood. When the sample flows through detection line 1, QD1-Ab1-PCT is captured by the fixed 14A2 mAb (Ab3 for short). QD1-Ab1-PCT-Ab3 is formed and fixed on the detection line 1 while the remaining sample keeps flowing without reacting. After that, when the sample keeps flowing through detection line 2, QD2-Ab2-CRP does not react with the fixed CRP, while extra QD2-Ab2 is captured by the fixed CRP and QD2-Ab2-fixed CRP is formed. The remaining sample including excessive QD1-Ab1 and uncombined QD2-Ab2-CRP keeps flowing through the quality control line, and part of it is captured by goat anti-mouse IgG (Ab4) fixed there. Therefore, there is a positive correlation between the fluorescent intensity on detection line 1 and the concentration of PCT, while there is a negative correlation between the fluorescent intensity on detection line 2 and the concentration of CRP. The direct observation of detection effect of the dual-QDs-labeled LFS is shown in Fig. 4.

### Limits of Detection

**Fig. 5:**
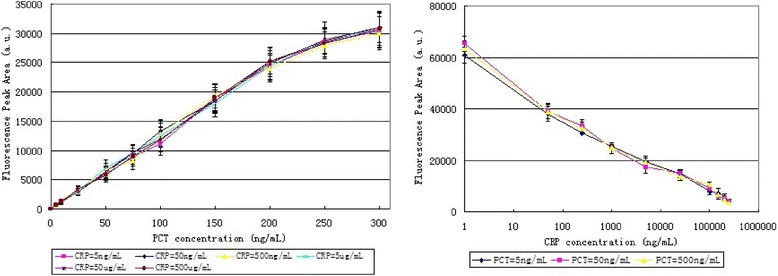
(*Left*) the calibration curve for detecting PCT, when fixed the CRP concentrations. (*Right*) the calibration curve for detecting CRP (*x*-axis is in log coordinates), when fixed the PCT concentrations. (*n* = 5)

For PCT, the minimum detection limit is set to be three times of the standard deviation of the blank control group’s background signal: SD0 (SD0 = 0.874, *n* = 5); the lower limit of quantitative detection is set to be 10 times of SD0; and the upper limit of quantitative detection is set to be the calibration curve’s turning point. As shown in Fig. 5 (left), for PCT, the quantitative detection range is between 0.3 and 200 ng/mL, and the minimum detection limit (i.e., sensitivity) is 0.1 ng/mL.

For CRP, since the inhibition method is adopted, the minimum detection limit of CRP is set to be the control group’s response T0 minus three times of the standard deviation SD0 (SD0 = 315.6, *n* = 5); the lower limit of quantitative detection to be the control group’s response T0 minus 10 times of the standard deviation SD0; and the upper limit of quantitative detection to be the total amounts of CRP fixed on detection line 2. As shown in Fig. 5 (right), for CRP, the quantitative detection range is between 50 ng/mL and 250 μg/mL, and the minimum detection limit (i.e., sensitivity) is 1 ng/mL.

### Calibration Curves, Interference, and Linearity

For PCT, the calibration curves of PCT were estimated with 11 different concentrations of standard PCT, from 0.2 to 300 ng/mL, diluted in PCT-free serum, when standard CRP was fixed at five different concentrations separately. The calibration curves of PCT were plotted in Fig. 5 (left). The maximum interference rate was 8.5 %, which is set to be the relative percentage between the maximum deviation and the mean value. The results indicated few evidences of interference by the concentrations of CRP. The calibration curves were linear fitted, as a reliable correlation coefficient (*R*^2^) was 0.9948, and a good linearity between fluorescence intensity and PCT concentration was *y* = 123.63*x* + 3.5259.

For CRP, the calibration curves of CRP were estimated with ten different concentrations of standard CRP, from 1 ng/mL to 250 μg/mL, diluted in CRP-free serum, when standard PCT was fixed at three different concentrations separately. The calibration curve of CRP was plotted in Fig. 5 (right). The maximum interference rate was 5.2 %, which is set as above. The results also indicated few evidences of interference by the concentrations of PCT. The calibration curves were also linear fitted, as a reliable correlation coefficient (*R*^2^) is 0.9838, and a good linearity between fluorescence intensity and the logarithmic CRP concentration is *y* = −4485.3Ln(*x*) + 59,131.

### Stability

The stability study of the dual-QDs-labeled LFS was conducted. The strips airtight preserved at 4 °C and the normal temperature were assayed at various time points (weeks 0, 2, 4, 6, 8). The standard solutions were prepared containing various concentrations of PCT (0.3, 20, 100, 200 ng/mL) and CRP (50 ng/mL, 5 μg/mL, 50 μg/mL, 250 μg/mL). The relative standard deviation is set to be relative percentage between the standard deviation and the mean value. It is found out that the strip still functions well for detection after 3-month airtight preservation at normal temperature, as the relative standard deviations for PCT and CRP were ±6.1 and ±8.4 %, respectively. Still, functions were well for detection after 4-month airtight preservation at 4 °C, as the relative standard deviations for PCT and CRP were ±5.3 and ±4.7 %, respectively.

### Contrasting Analysis with Detection Results by Existent Equipment

**Fig. 6:**
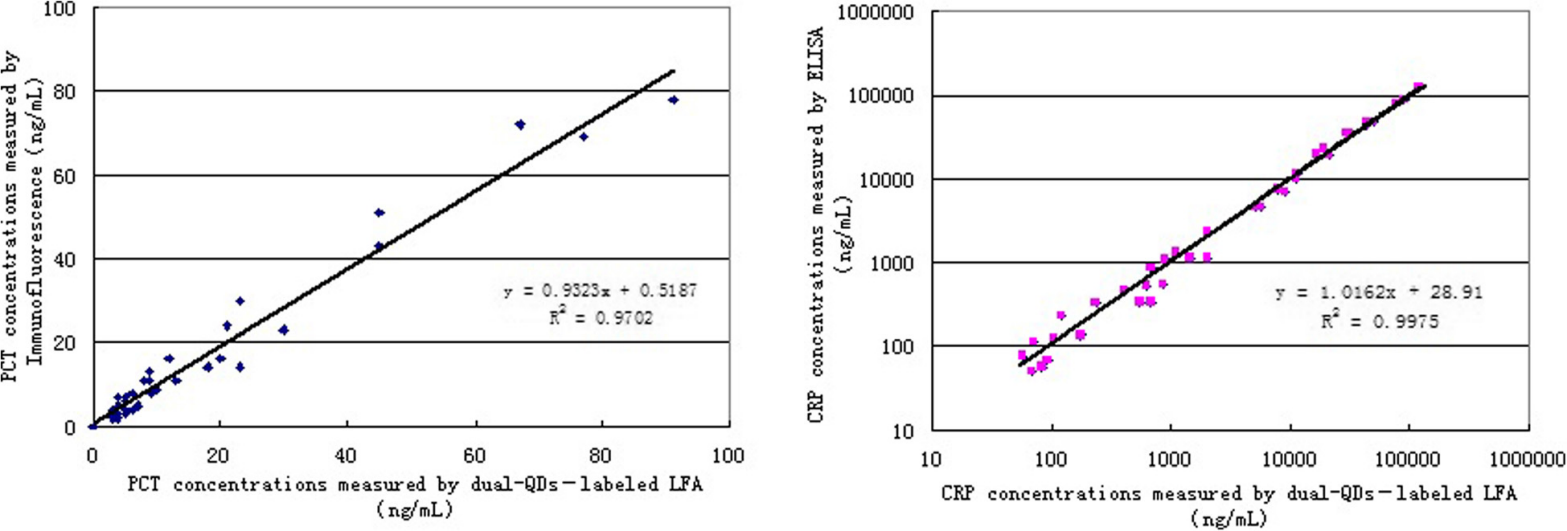
(*Left*) comparing testing results of PCT concentrations by our method and immunofluorescence. (*Right*) comparing testing results of CRP concentrations by our method and ELISA, shown in double-log coordinates. (*n* = 3)

The correlations between the detection results by the hospital and by the dual-QDs-labeled LFS method established in this paper were shown in Fig. 6. Correlations for CRP and PCT were 99.75 and 97.02 %, respectively, which indicated that the detection results of PCT by our method and immunofluorescence are consistent and also indicated the same of CRP by our method and ELISA. These results mean that the dual-QDs-labeled LFS method for CRP and PCT has a good performance compared with other widely commercialized methods.

## Discussion and Conclusions

In this paper, hydrophobic CdSe/ZnS QDs with two different emission spectra were prepared first and modified by 14C12 mAb and CRP135 mAb, respectively, to produce QD-antibody conjugates. The QD-antibody conjugates were sprayed on the LFS. Furthermore, a double antibody sandwich method for PCT while a competitive inhibition method for CRP was employed in the LFS. Accordingly, the corresponding fluorimetry system is designed for detecting the fluorescence intensity on detection lines 1 and 2 on the test strip. By testing with standard samples and clinical samples, the characteristics of QDs and dual-QDs-labeled LFS were analyzed.

There are various advantages of using dual QDs as the markers in LFS [30]. First, the sensitivity is at least 1 to 2 magnitudes higher than that of colloidal gold, which is commonly used in LFS. The concentration of PCT in the blood which can be clinically explained is between 0 and 10 ng/mL. This scope cannot be distinguished by the existing commercial LFS. While PCT sensitivity detected by the dual-QDs-labeled LFS method established here reached 0.1 ng/mL, which was consistent with the sensitivities detected by immunofluorescence method and immunochemiluminescence. Second, spectra emitted by QDs have a narrow spectral peak. Besides, the fluorescence spectra can be received by CCD and processed digitally. In this article, two QD-antibody conjugates with different emission spectra were produced and employed in the LFA to detect PCT and CRP simultaneously. There is little interference from the external lights, and little interference between PCT and CRP assay. Furthermore, 20 min is enough for testing the PCT and CRP concentrations in the whole blood by the dual-QDs-labeled LFS, since blood cells can be filtered by the whole blood separating membrane and those that are not filtered move very slowly.

By testing the standard samples and clinical samples, it was confirmed that the concentrations of PCT and CRP in the blood can be simultaneously quantified by dual-QDs-labeled LFS method designed in this paper. For PCT assaying, the quantitative detection range was from 0.3 to 200 ng/mL and the sensitivity was 0.1 ng/mL, while for CRP assaying, from 50 ng/mL to 250 μg/mL and 1 ng/mL, respectively, which are comparable to the characteristics of the existences. Besides, the step of serum separation is required in the existing methods. The dual-QDs-labeled LFS has comparative advantages in that firstly, whole blood can be detected by it without requirement of serum extraction; secondly, simultaneous quantification of CRP and PCT can be conducted within 20 min by using only 100 μL of the blood; and thirdly, its paired equipment for fluorescence detection is simple to operate and also at a low cost.

However, there are still several problems in the dual-QDs-labeled LFS designed in this paper. Firstly, the waste antibodies in producing QD-antibody conjugates caused the strip at a high cost, due to the low valence of QDs and the price of monoclonal antibodies. Secondly, to minimize the hook effect, competitive inhibition method employed to detect CRP in the dual-QDs-labeled LFS caused the quantifying limit not low enough to detect the contents of high-sensitivity CRP (hs-CRP). Thirdly, the dual-QDs-labeled LFS method needs to be applied in a temperature-humidity controlled environment or calibrated frequently, since the fluorescence intensity signal is easily affected by environmental factors.

Nevertheless, dual-QDs-labeled LFS established in this paper can simultaneously quantify PCT and CRP contents in human blood just in several minutes without sample pretreatment. It has great value for determining the severity and prognosis of bacterial infection and providing guidance for the usage of antibiotics, especially in the case of emergency or field assay. Furthermore, it also can help assay some given diseases [1, 2, 11].
